# Early-Life Sleep Deprivation Enhanced Alcohol Consumption in Adolescent Rats

**DOI:** 10.3389/fnins.2022.856120

**Published:** 2022-04-25

**Authors:** Fatin Atrooz, Ghalya Alrousan, Arham Hassan, Samina Salim

**Affiliations:** Department of Pharmacological and Pharmaceutical Sciences, University of Houston, Houston, TX, United States

**Keywords:** sleep deprivation, alcohol consumption, adolescents, reward, early life stress

## Abstract

Evidence in the literature suggests that sleep deprivation during early-life developmental stages, by impacting important processes such as the reward circuit maturation, may increase the vulnerability for alcohol and substance use. The mechanisms involved are not fully understood. In this study, utilizing our previously established model, we examined the impact of early-life sleep deprivation on alcohol consumption in adolescent rats. Male Sprague Dawley rats served as either the control (CON) or sleep-deprived (SD) group. Sleep deprivation was induced using a Pinnacle automated sleep deprivation apparatus. The SD group of rats was sleep deprived for 6–8 h/day for 14 days from postnatal day (PND)19 to PND32. At PND33, anxiety- and depression-like behaviors were assessed in rats using elevated plus maze and sucrose splash test, respectively. At PND39, alcohol consumption was assessed in rats for five consecutive days using the two-bottle choice paradigm, water versus 5% ethanol. SD rats exhibited significant anxiety- and depression-like behaviors as compared to CON rats. Interestingly, SD rats consumed a larger volume of alcohol when compared to CON rats, which was significantly higher at day 5 (mean of alcohol consumption (ml) ± SD; CON = 6.67 ± 3.42; SD = 19.00 ± 6.05, *p* = 0.0126). SD rats also showed high preference for alcohol over water, which was significantly higher at day 5 (mean of alcohol preference (%) ± SD; CON = 26.85 ± 14.97; SD = 57.69 ± 5.61, *p* = 0.014). Our data suggest that early-life sleep deprivation enhanced alcohol consumption in adolescent rats.

## Introduction

It is now well known that modern-day children and adolescents get less sleep than the kids in the previous generations (Dollman et al., 2007). The American Academy of Sleep Medicine recommended that adolescents should sleep 8–10 h per night to promote optimum health (Paruthi et al., 2016), but large survey studies conducted by the Youth Risk Behavior Surveys (YRBSs) reported that around 72% of adolescents sleep less than the recommended sleep duration (Wheaton et al., 2018). Excessive use of electronic devices, extended use of screen time at night, early school time, and large school workload have largely contributed to shorter duration of sleep in today’s youth (Hale and Guan, 2015; Woods and Scott, 2016; Gariepy et al., 2020). The reduced weekday sleep time and oversleep over the weekend, to make up for lost sleep during the weekday, are postulated to result in chronic sleep deprivation or “social jetlag” (Seton and Fitzgerald, 2021). The consequences of chronic sleep deprivation on stress response, mood, anxiety, and cognition have been investigated in preclinical (Pires et al., 2012, 2016; Abel et al., 2013; Hirotsu et al., 2015; Villafuerte et al., 2015; Atrooz et al., 2019) and epidemiological settings (Goel et al., 2013; Minkel et al., 2014). Lately, insufficient sleep in adolescent and young adults has been associated with risk behavior and substance use including alcohol, marijuana, and cigarettes (Wong et al., 2010, 2015; Hasler et al., 2012; Kenney et al., 2012; Logan et al., 2018; Kwon et al., 2019, 2020; Hasler and Pedersen, 2020). Sleep deprivation, especially in adolescents, negatively impacts normal behavior, emotion, and attention (Dahl and Lewin, 2002). This seems reasonable as adolescence is a sensitive developmental period and often highly susceptible to anxiety and depression (Costello et al., 2003; Barker et al., 2019), as well as risky behavior such as substance use and abuse (Young et al., 2002; Peiper et al., 2016). The prevalence of adolescent substance use has rapidly increased particularly in the past three decades (Chassin et al., 2004). Alcohol is one of the most commonly used substances among adolescents, followed by marijuana (Gray and Squeglia, 2018).

The adolescence phase is characterized by increased reward-seeking behavior. The brain undergoes significant neurodevelopmental changes occurring between the childhood phase and young adulthood period, which continues until the age of 25 (Pfefferbaum et al., 1994; Giedd, 2004). Human and animal studies both have suggested differential developmental trajectories of limbic reward systems relative to top-down prefrontal control systems during adolescence, with limbic systems developing earlier than prefrontal control regions. According to this model, in emotionally salient situations, the limbic system predominates the prefrontal control systems, thus increasing the engagement in risky behavior during adolescence (Casey et al., 2008). A growing body of evidence suggests that poor sleep may exaggerate the normative maturation imbalance between affective and cognitive control systems, leading to greater risk-taking behaviors in adolescents (Kenney et al., 2012; Telzer et al., 2013), including substance use (McKnight-Eily et al., 2011), yet the underlying mechanism(s) affording such behaviors are not fully understood. Therefore, examining the neurobiological underpinnings which may connect sleep deprivation and substance use during adolescence will be critical for appropriate intervention.

In this study, utilizing our previously established model (Atrooz et al., 2019), we examined the impact of early-life sleep deprivation on alcohol consumption in adolescent rats. We hypothesize that early-life sleep deprivation will increase the reward-seeking behavior in adolescent rats indicated by enhanced voluntary alcohol intake. Male Sprague-Dawley rats at postnatal day (PND) 19 served as either the control (CON) or sleep-deprived (SD) group. Sleep deprivation was induced using a Pinnacle automated sleep deprivation apparatus. The SD group of rats was sleep deprived for 6–8 h/day for 14 days from PND19 to PND32. This developmental period in rats represents childhood and early adolescence stages, which is considered comparable to 2–11 years in human age (Semple et al., 2013). We observed that alcohol consumption increased in rats that were exposed to early-life sleep deprivation as compared to control rats. This study forms the premise for future studies designed to explore the structural and functional determinants of the reward centers of the brain, which may reveal biochemical triggers that connect early-life sleep deprivation with increased reward-seeking behavior and enhanced alcohol consumption in adolescents.

## Materials and Methods

### Sleep Deprivation Protocol

All experiments were conducted in accordance with NIH guidelines using approved protocols from the University of Houston Animal Care Committee. A sleep deprivation protocol was conducted as described in our previous publication (Atrooz et al., 2019). Consolidated litters of lactating Sprague Dawley female with male pups with mothers were purchased from Envigo, United States. The pups with dam arrived at our animal facility at PND11. The rats were acclimatized in the animal facility for 7 days with a 12 h light/12 h dark cycle, lights on at 7:00 am. At PND18, the pups were separated from their mothers and provided with water gel and food *ad libitum*. Two rats were housed per cage to eliminate isolation stress; at PND19, the rats were randomly assigned into two groups: SD and CON groups, with eight rats per group. The SD rats were subjected to sleep deprivation 6 h/day starting at 8:00 am for 7 days; two rats were placed in each sleep deprivation apparatus to exclude social isolation stress. After 7 days, the sleep deprivation duration was increased to 8 h/day for seven additional days as the percentage of wakefulness during the light cycle increases with development in rats (Alfoldi et al., 1990). The sleep deprivation was induced using a Pinnacle automated sleep deprivation apparatus (Pinnacle Technology, Lawrence, KS, United States). The apparatus is composed of a Plexiglas cylindrical cage with a rotating bar at the base. The bar is controlled by a software (Sirenia Acquisition) which enables controlled rotation to maintain the desired speed and direction. Random bar rotation was selected to prevent adaptation to bar rotation at speeds of 10–40 rotations per minutes, so the rotating bars gently touch the rats’ feet and disturb their sleep. The cages were layered with corn cob bedding and equipped with water bottles. Chow pellets were cut into small pieces and provided *ad libitum*. The rats were continuously observed by the experimenter throughout the sleep deprivation protocol. This sleep deprivation system effectively induces sleep deprivation in rats as validated by polysomnography (Hines et al., 2013; Wooden et al., 2014). Moreover, we used a non-invasive method to estimate the sleep/wakefulness pattern in rats during and after the sleep deprivation protocol. This method is based on analyzing the activity of the rats in their home cages through continuous video acquisition. Estimation of a sleeping phenotype depends on the continuous recording of rat’s activity/inactivity; the longer the rat stays inactive, the more likely the rat is asleep (Pack et al., 2007). We found that SD that rats showed continuous activity during sleep deprivation hours suggested a wakefulness phenotype, while control rats showed a sleeping phenotype (Atrooz and Salim, 2020). While this method of sleep deprivation assessment lacks detection of micro-sleep bouts and rebound sleep, it allows high-throughput measurement of sleep phenotype in rats without the need for surgery and chronic electrode implantation.

The control rats were placed in similar cages (two rats per cage) and left undisturbed in the same room. One day following the conclusion of the sleep deprivation protocol; at PND33, behavior tests were conducted as described in the following sections.

#### Anxiety-Like Behavior Assessment

Anxiety-like behavior was examined using the elevated plus maze (EPM) test: The EPM apparatus consisted of two open and two closed arms (10 cm × 50 cm) that intersected to create a plus shape at an elevation of about 60 cm from the ground. Each rat was placed in the intersection area facing the open arms of the maze and allowed to explore the maze. Movement of the rats between the arms was recorded for 5 min and analyzed using the AnyMaze software as previously published (Salim et al., 2011; Vollert et al., 2011). Anxiety-like behavior in rats was indicated by rats spending less time in the open arms.

#### Depression-Like Behavior Assessment

Depression-like behavior was assessed using the sucrose splash test. The rat grooming behavior following spraying the sucrose solution on the dorsal coat reflects self-care and motivational behavior (Lefter et al., 2018). The sucrose splash test was performed in the open-field apparatus. Each rat was splashed two times with 10% sucrose solution on the dorsal coat using an atomizer spray bottle. After applying the sucrose solution, the total time spent in grooming, the latency to the first bout of grooming, and the number of grooms within a 5 min test period were recorded as indices of depression-like behavior (Ortmann et al., 2016; Hu et al., 2017; Lefter et al., 2018).

### Voluntary Alcohol Consumption

One week following the conclusion of the SD protocol, at PND39, alcohol intake was measured in rats for five consecutive days (PND39–PND43). We opted for a 1 week wait period before alcohol intake assessments were made because sexual maturity and impulsivity behaviors in rats manifest between PND35 and PND49 (Semple et al., 2013; Sengupta, 2013). Another consideration for inclusion of the wait period was to allow the rats to recover from sleep deprivation before assessing their voluntary alcohol intake, thus eliminating any confounding factors that may arise from direct influence of sleep debt on alcohol consumption. Alcohol intake was assessed in rats using the two-bottle choice paradigm, water versus ethanol. In this paradigm, rats had free access to two identical bottles in their home cages, one filled with 300 ml alcohol (5%) and the other filled with 300 ml water. Alcohol solution (5%) was prepared from 96% ethanol (Everclear Grain Alcohol 190) diluted in tap water. We used 5% alcohol because it is similar to the alcoholic beverages that are mostly consumed by adolescents (Wille-Bille et al., 2017). The positions of the water and alcohol bottles were alternated daily to eliminate any bias toward the position of the bottles.

### Assessment of Alcohol Consumption

Alcohol and water intake was measured daily at 11:00 am, and the bottles were refilled with the corresponding fluids. Since two rats were housed per cage, the quantity of fluids consumed was divided by 2. Alcohol preference = [volume of alcohol intake (ml)/total fluid intake (water + alcohol) (ml)] × 100%.

### Statistical Analysis

First, all data were assessed for normality of distribution using the Kolmogorov–Smirnov test. Data are expressed as mean ± standard deviation (SD). Behavior data were analyzed using the Student *t*-test. Alcohol consumption and alcohol preference across the five experimental days were analyzed using a two-way ANOVA test. *P*-values < 0.05 were considered statistically significant. Data were analyzed using GraphPad Prism 9.1 statistical software (GraphPad Software San Diego, CA, United States).

## Results

**FIGURE 1:**
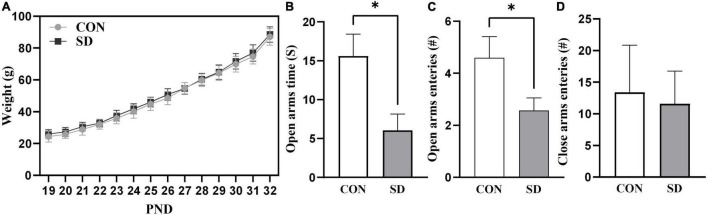
Growth curve and anxiety-like behavior. Rats’ weight was measured daily throughout the sleep deprivation protocol **(A)**. Lines represent the means of weight in grams (g) ± SD. Rats were assessed for anxiety-like behavior at PND33 using EPM test, time spent in open arms **(B)**, number of open-arm entries **(C)**, and number of close-arm entries **(D)**. Bars are means ± SD, *n* = 6–8 rats per group. Data were analyzed using two-tailed unpaired *t*-test. (^∗^) Significantly different at *p* < 0.05. PND: Postnatal day. Group designations: control (CON), sleep deprivation (SD).

Rats in the SD group exhibited a normal-weight-gain pattern during the sleep deprivation protocol. Weight gain pattern in SD rats was comparable to CON group suggesting that sleep deprivation protocol did not affect body weight-gain and growth in rats, (see Figure 1A).

### Anxiety-Like Behavior

The EPM test measures anxiety-like behavior based on rodents’ aversion for elevated and open areas (Patki et al., 2015). Less time spent in the open arms of the EPM test apparatus is an indication of increased anxiety-like behavior. In the EPM test, the SD rats spent significantly less time in the open arms as compared to CON rats, *t*(10) = 2.71, *p* = 0.022 (Figure 1B). The SD rats also showed less number of entries into the open arms as compared to CON rats, *t*(10) = 2.29, *p* = 0.045 (Figure 1C). Importantly, there were no significant differences between SD and CON rats in the number of entries to the close arms, *t*(10) = 0.50, *p* = 0.625 (Figure 1D), suggesting that there was no difference between the group in general activity/locomotion. The EPM data suggested that SD exhibited anxiety-like behavior, when compared with CON rats.

### Depression-Like Behavior

**FIGURE 2:**
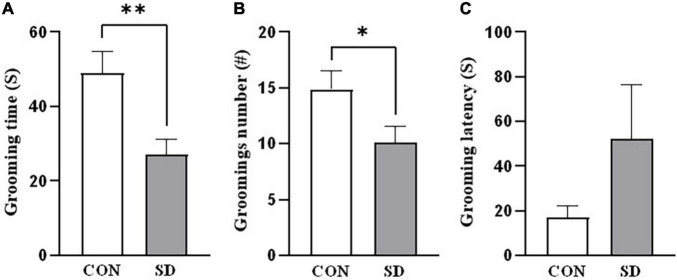
Depression-like behavior. Depression-like behavior was assessed in rats using the sucrose splash test. The time the rats spent grooming **(A)**, the number of grooming sessions **(B)**, and the latency to start the first grooming session **(C)**, following splashing of the dorsal coat with 10% sucrose. Bars represent means ± SD. Data were analyzed using *t*-test. (^∗^) Significantly different from control at *p* < 0.05. *n* = 6–8 rats per group. (^∗∗^) Significantly different from control at *p* < 0.001. Group designations: control (CON), sleep deprivation (SD).

In splash test, after spraying the rats with 10% sucrose, we investigated the grooming time and the number of grooming sessions as indicators of self-care behavior. SD rats spent less time grooming when compared to CON rats [grooming time: *t*(14) = 3.02, *p* = 0.009; number of grooming sessions: *t*(14) = 2.19, *p* = 0.046] (Figures 2A,B). However, latency to the first grooming session was comparable in SD and CON rats, *t*(14) = 1.44, *p* = 0.173 (see Figure 2C).

### Alcohol Consumption

**FIGURE 3:**
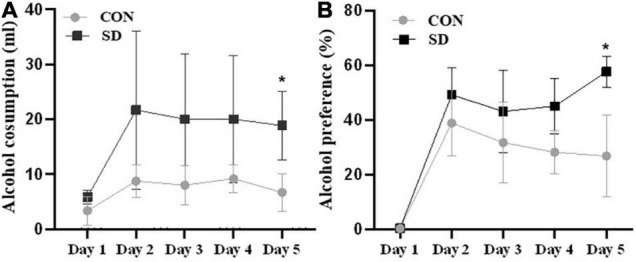
Alcohol consumption. Alcohol consumption was assessed in rats using two-bottle choices, water versus 5% alcohol. The average volume of alcohol intake per day in milliliter (ml) **(A)** and the percentage of alcohol preference over water **(B)** were assessed for five consecutive days (PND39–PND43). Bars represent means ± SD. Data were analyzed using *t*-test, *n* = 6 rats per group. (*) Significantly different from control at *p* < 0.05. Group designations: control (CON), sleep deprivation (SD).

Rats at the adolescent stage (PND39) were assessed for voluntary alcohol consumption daily for five consecutive days. Interestingly, a two-way ANOVA analysis showed a significant effect of group on alcohol consumption [*F*(1, 10) = 7.50), *p* = 0.021]. Data also showed a significant effect on alcohol consumption [CON-SD; *F*(4,40) = 11.70, *p* = 0.005] and interaction between day and group [*F*(4, 40), *p* = 0.035]. A pairwise multiple comparison revealed significant enhancement in alcohol consumption in SD rats as compared to CON rats on day 5 [mean of alcohol consumption (ml) ± SD; CON = 6.67 ± 3.42; SD = 19.00 ± 6.05, *p* = 0.0126] (see Figure 3A). Sleep deprivation also showed a significant effect on alcohol preference as revealed by a two-way ANOVA [*F*(1, 10) = 8.49, *p* = 0.015], day effect was also significant [*F*(4, 40) = 37.13, *p* < 0.0001], and the interaction between day and group was significant [*F*(4, 40) = 7.08, *p* = 0.0002]. Pairwise multiple comparison revealed that SD rats showed significantly higher preference for alcohol as compared to CON rats on day 5 [mean of alcohol preference (%) ± SD; CON = 26.85 ± 14.97; SD = 57.69 ± 5.61, *p* = 0.014] (see Figure 3B).

## Discussion

In this study, we observed that early-life sleep deprivation enhanced alcohol consumption in adolescent rats. This was accompanied with anxiety- and depression-like behaviors in adolescent rats. To our knowledge, our study is the first preclinical model to report the enhancement effect of early-life sleep deprivation on alcohol consumption in adolescent rats. Only one group studied the impact of chronic partial sleep deprivation on alcohol consumption in adolescent rats. The authors reported enhancement of alcohol consumption following sleep deprivation. However, control conditions were also associated with increased alcohol consumption. Hence, the authors could not conclude that there is a causal relationship between sleep deprivation and alcohol consumption (Sequeira, 2015; Khan, 2021). On the other hand, several rodent studies have examined the relationship between sleep deprivation, alcohol consumption, and behavior in adult animals (Aalto and Kiianmaa, 1984; Cowan, 2017). One study conducted in adult rats showed that sleep restriction (4 h/day for 7 days) stimulated alcohol consumption and induced a significant increase in the number of delta FosB-positive cells in the NAc and VTA brain regions, suggesting the increased activity of reward-related circuitry of the brain following prolonged sleep restriction (García-García et al., 2021). Another study conducted in adult rats suggested that REM-sleep deprivation increases alcohol intake and alcohol consumption, which further intensifies REM-sleep deprivation (Aalto and Kiianmaa, 1984). This is not surprising as alcohol is believed to alter sleep homeostasis, inducing insomnia and sleep disruption. Six weeks of ethanol exposure in rats produced persistent diminution in cortical slow-wave power (Ehlers and Slawecki, 2000). Acute sleep deprivation in mice induced a sustained impairment in adenosine-regulated sleep homeostasis, which lasted for 4 weeks and altered the sensitivity to the motor-impairing effects of alcohol (Clasadonte et al., 2014). The results suggested that chronic sleep restriction induced a sustained impairment in adenosine-regulated sleep homeostasis and consequentially impacted the response to alcohol.

Our data suggesting that early-life sleep deprivation leads to anxiety-like and depression-like behaviors in adolescent rats are significant and translationally relevant, as the relationship between affective problems and substance use has been established in several human studies (Valentiner et al., 2004; Wu et al., 2010). Affective problems, such as depression and anxiety, which often emerge during adolescence, are reported to be associated with greater rates of substance use. In fact, chronic sleep deprivation in children and adolescents is believed to increase the risk for mood and behavioral problems such us drug and alcohol use (Johnson and Breslau, 2001; Smaldone et al., 2007; Wong et al., 2010, 2015; Logan et al., 2018). Poor sleep during early life prospectively predicted alcohol-related problems and illicit drug use in adolescents and young adults (Wong et al., 2010, 2015). In a sample of South Korean middle- and high-school students aged 13–18 years, self-rated stress levels and depression were inversely proportional to nocturnal sleep duration. Health risk behaviors such as smoking and alcohol consumption were more common in sleep-deprived students (Lee et al., 2013). Adolescents’ preference for later activity and sleep is associated with greater alcohol involvement (Pieters et al., 2010; Urbán et al., 2011). Furthermore, delayed sleep time was associated with negative outcomes such as lower average school grades, smoking, alcohol use, and elevated anxiety and depression scores in Norwegian adolescents (Saxvig et al., 2012).

Finally, our data are important as they imply that sleep deprivation during developmental stages of early life may be a risk factor for alcohol use in adolescents. A growing body of evidence suggests that exposure to alcohol at early age increases the risk of alcohol dependence and drug addiction in adolescent and young adults (Grant et al., 2006; Hingson et al., 2006). In a 6-year cohort study, alcohol dependence in young adults was preceded by higher persisting teenage rates of frequent drinking (Bonomo et al., 2004). In adolescent rats, intermittent ethanol treatment induced short- and long-lasting cognitive and behavioral deficits and caused prefrontal cortex and hippocampal damage by inflammatory processes (Pascual et al., 2007). The sensitivity of adolescent brain to ethanol exposure involved dopaminergic and glutamatergic neurotransmission, leading to long-lasting abnormal plasticity in reward-related brain regions that could contribute to alcohol addiction (Pascual et al., 2009).

## Future Directions

This study is a brief report in which we have established a preclinical model for studying the impact of sleep deprivation on alcohol consumption in adolescent rats. Several aspects are considered for future studies. First, we are interested in examining sex-based differential behavioral responses to early-life sleep deprivation in adolescent rats. Second, understanding resilience and susceptibility to developing dependence, reward-seeking behavior, and behavioral impairments using correlation analysis is a future aim of this project. Third, clarifying brain region selectivity and changes in brain circuitry which controls reward-seeking behavior and associated with enhancement of alcohol consumption is a future aim of this study. This will be conducted using imaging, molecular, and electrophysiological methodologies.

## Data Availability Statement

The original contributions presented in the study are included in the article/supplementary material, further inquiries can be directed to the corresponding author/s.

## Ethics Statement

The animal study was reviewed and approved by Institutional Animal Care and Use Committee (IACUC), University of Houston.

## Author Contributions

FA and GA conducted the sleep deprivation protocol and the behavior assessment experiments. AH and GA conducted the data analysis. FA wrote the first draft of the manuscript. SS finalized the draft after several layers of edits and iterations by all authors.

## Conflict of Interest

The authors declare that the research was conducted in the absence of any commercial or financial relationships that could be construed as a potential conflict of interest.

## Publisher’s Note

All claims expressed in this article are solely those of the authors and do not necessarily represent those of their affiliated organizations, or those of the publisher, the editors and the reviewers. Any product that may be evaluated in this article, or claim that may be made by its manufacturer, is not guaranteed or endorsed by the publisher.
